# A Report of One Hundred and Sixty Abdominal Sections, with Special Reference to the Prevention of Unpleasant Sequelæ1Read to the Buffalo Medical and Surgical Association, February 11, 1890.

**Published:** 1890-04

**Authors:** Matthew D. Mann

**Affiliations:** Professor of Obstetrics and Gynecology, Medical Department, University of Buffalo; 31 Allen Street


### A REPORT OF ONE HUNDRED AND SIXTY ABDOMINAL SECTIONS, WITH SPECIAL REFERENCE TO THE PREVENTION OF UNPLEASANT SEQUELÆ.[1]

By MATTHEW D. MANN, A. M., M. D.

Professor of Obstetrics and Gynecology, Medical Department, University of Buffalo.

FROM the time of my first operation in Buffalo, January 4, 1882, to January 4, 1890, a period of eight years, I have opened the abdomen 160 times.

The cases can be classified as follows:

Removal of ovarian tumors, one hundred, with thirteen deaths.
" " fibroid tumors alone, (myomotomy,) six, with one death.
" " uterus for fibroids, (supra-vaginal hysterectomy,) five, with no deaths.
" " diseased tubes and ovaries, fourteen, with one death.
" " diseased ovaries, sixteen, with no deaths.
" " ovaries for uterine fibroids, two, with no deaths.
" " fibro-cyst of uterus, one, recovery.
Exploratory, eight, with no deaths.
For causes outside of the pelvis, eight, with two deaths.

I make a class of cases for objects outside of the pelvis, because they are cases which have only fallen into my hands by accident, or through necessity. I design confining myself strictly to pelvic diseases in women, and therefore separate these cases from the rest, as not belonging strictly to my specialty.

The cases were as follows:

Resection of intestine, one, death.
Obstruction of bowels, one, recovery.
Exploratory, one, death.
Abscess, two, recoveries.
Exploratory, cancer of omentum, one, recovery.
Tumor of abdominal wall, one, recovery.
Tubercular peritonitis, one, recovery.

The deaths were distributed as follows: In the first fifty—eleven, in the second fifty—five, and in the last sixty[2]—one death.

Total deaths, seventeen, or a little over ten per cent.

What stronger argument could there be than these figures to show the advantage of experience. In the first 100, sixteen deaths, and in

1. Read to the Buffalo Medical and Surgical Association, February 11, 1890.
2. This number has now reached seventy seven.

the second 100, so far, one. Still, a careful examination of the cases would show that neither increased experience nor could anything else have saved some of the earlier cases. One died from heart clot on the third day—three from shock, one from long existing chronic peritonitis, a month after operation. Two were far gone in septicemia from suppuration of the cyst. One died from obstruction of the bowels, several weeks after operation. Eight of the seventeen cases died from sepsis, including the two who were septic before operation. It is in the avoidance of this complication that the greatest advance has been made, there having been but two cases of septicemia in the last 120 cases. The single death in the third series of sixty cases was from obstruction of the bowels.

As regards the character of the cases operated on, it will be noticed that as compared with the reports of many other operations, there is a preponderance of operations for tumors (118) and a relatively small percentage of removal of ovaries, or tubes and ovaries.

I have been very conservative in this regard, and have frequently refused to operate on ovaries which I have been fairly importuned to remove, because I saw no good reason either in the symptoms or in the physical evidence of disease. Other cases have been operated on only after years of treatment—often in my own hands—has failed to do the slightest good.

When these cases are properly selected they are among the most satisfactory imaginable. I have followed up all the cases, where it was possible, and in nearly every instance the cure has been complete. To convert a life-long invalid into a healthy, useful member of society is certainly a satisfaction, and such has been the result in many instances. It is not the place and time to detail cases, but some of those on the list are of extreme interest, and will, I hope, form the subject for a paper at some future day.

There is a smaller and decreasing proportion of uterine fibroid tumors operated upon—this in the face of the fact that the results have been exceedingly satisfactory ; only one death in thirteen operations involving fibroids. The reason is to be found in the excellent results which have been and are being attained in the treatment of these cases by galvanism. Only occasionally does a case turn up which seems proper for operation.

Now to come to the second part of my paper—the avoidance of unpleasant sequelæ after abdominal section.

Until recently surgeons have been occupied, especially in this country, with the idea that in abdominal surgery the main point is to save the patient's life regardless of after complications.

The wonderful results attained by European surgeons, as compared with those obtained in this country, have forced American surgeons to a higher effort to improve their own records, and in this endeavor other points of nearly as great importance have been well-nigh lost sight of.

Of what advantage is it to remove a woman's tubes and ovaries, or even a tumor which itself may not be endangering life, if, in place of the disease, we leave another condition nearly as bad? Lately, thanks to the perseverance and growing skill of our operators, the statistics of abdominal surgeons in this country have been placed on a par with those of our European compeers. To-day no woman need go to Europe for the sake of improving her chances in such an operation.

It has been found that the bad results formerly obtained were not due to the peculiar nervous condition of our American women, as was often asserted, or to climatic influences, or anything except the want of proper methods. With improvement in these methods and added experience, no reason existed, or exists, why we should not be able to save as many here as in any other part of the world—and this is now abundantly proved by the facts. Laparatomy in America is now, in the hands of our best operators, as safe as any capital operation can be. This much, then, having been accomplished, it is time that we look further, and try to reduce to a minimum those unpleasant sequelæ, which, while not directly endangering the life of the patient, often render life less enjoyable, and sometimes lead to its ultimate unnecessary loss. The note has already been sounded, and the attention of operators has been, of late, frequently called to these unpleasant results.

The first of the sequelæ, to which I will ask your attention, is the production of a ventral hernia. It must be the experience of every one who has often opened the abdomen, to have his patients after a time return, complaining of more or less of a hernial protrusion in the line of the incision. Although it may be supported by a pad, truss, or abdominal bandage, it is certainly a very great annoyance to the patient, and, as I have lately observed, a source of danger. Quite recently I was called to see Mrs. B., with the somewhat startling statement that she had burst open. In June, 1888, I removed from this patient two very large ovarian tumors, which had existed from fifteen to sixteen years. The operation was very severe. The adhesions were extensive and very dense, and the smaller tumor had developed between the folds of the broad ligament. There was a very large oozing surface, and a drainage-tube was put in. She made a good recovery, and in time came back to know what she should do with a large ventral hernia which had first appeared at the former site of the drainage-tube, and had gradually grown upward, until a large portion of the long scar had been opened up, the skin and peritoneum only remaining intact. The patient had gained in flesh just about the weight of the tumor, viz.: sixty pounds. I advised her to wear an abdominal supporter, and if that did not do, to have the hernia operated on.

On the evening before I was recently called, on removing her supporter, she leaned over to untie her shoes, when the hernial sac ruptured, and like the man in Scripture, all her "bowels gushed out." Fortunately, Dr. Hanley was called, who washed off the protruding intestines with an antiseptic solution and returned them to the abdomen, holding them in place with an antiseptic pad and long adhesive strips. When I saw her in the morning she was perfectly comfortable—no shock or pains, and no fever. I at once removed her to the Hospital and operated. The operation was very long and tedious, as I was obliged to lay open the whole of the old incision, and to include the navel, where a hernial protrusion was beginning. I freshened the edges of the fascia, and, having brought it together with continuous catgut sutures, closed the whole with silver wire. She made a good recovery, though she nearly died from shock after this second operation. She was sixty-three years old. This case proves that a ventral hernia has, added to the risk of strangulation inherent in nearly all hernias, the risk of rupture.

How can this complication be avoided? Our anatomists tell us that the real supporting layer of the abdominal walls is the fascia. If this be in any way opened, the remaining structures of skin, fat, connective tissue and peritoneum, are utterly useless to withstand continuous pressure. Nor is scar tissue, unless it be keloid in its character, any better. Any one who depends on scar tissue as a supporting structure will, in the end, be disappointed. Although firm, and apparently strong, at first, it becomes weaker and softer with age, and finally affords no support at all. There are three ways in which the integrity of the fascia may be interfered with. First, by failure to secure perfect apposition of the edges in closing the wound. Second, by its separation by a drainage-tube, with subsequent cicatrization of the opening left; and, lastly, by a mural abscess forming below it, and opening it by its growth and subsequent rupture. How can this be avoided?

To secure perfect apposition I always make a point of carefully suturing, with continuous catgut sutures, the cut edges of the fascia. In this way, and in this way only, I maintain, can perfect apposition be ensured. In order to prevent with certainty septic infection of the tissue under the fascia, antiseptic irrigation is necessary. This can only be properly done after the abdominal cavity is closed. I, therefore, as the first step, carefully close the peritoneum with continuous catgut sutures, uniting the whole with silver wire placed entirely through the flaps. I must put in a little aside at this point. How wonderfully our ideas have changed regarding the peritoneum. In lately reading an account of his first successful ovariotomy, by Dr. Miner, I noticed that he attributed his success to the fact that he had not put any of his sutures through the peritoneum.

As to the use of the drainage-tube, I will only say that I use it very little, and then remove it as soon as possible. The dictum of somebody, "When in doubt—drain," has, I am sure, done more harm than good. I would modify the rule in this way: "When in doubt, pack the pelvis with sponges and wait." In this way drainage may often be dispensed with, and I have completely closed the cavity after packing and waiting with what seemed, at first, a very large, freely oozing surface. By the method of withholding fluids from the stomach after the operation, the peritoneum is forced, or, rather, stimulated to take up any fluid which may be effused. This accomplishes the same thing as drainage, and depending on it, and on previous packing, I have often left out the drainage-tube where others, doubtless, would have drained. I have never regretted not having drained. I have several times, been sorry that I had done it, and this brings me to my second point.

The production of a fistula or sinus, leading down deeply into the abdominal cavity, is a source of great annoyance to the patient, and is not without its dangers. These sinuses form in two ways. Either they result from an abscess which has gathered deeply within the pelvis and broken through the abdominal wound—as once happened to me—or they come through the use of a drainage-tube.

To prevent them, careful antisepsis on the one hand, and sparing use of the drain on the other, are necessary. But even if formed they usually heal unless they have some foreign substance at the bottom to present. This foreign body is generally a silk ligature. It was an experience of this kind—a fistula, which has lasted four years, and which has resisted all attempts to heal it, and which is doubtless kept open by a silk ligature at the bottom—which caused me to give up silk.

I have used no silk in the abdomen for the last 125 cases, using only catgut, which I prepare myself. The objections usually urged against catgut, I consider, groundless. I have left over 300 pieces of catgut within the abdomen and have never seen the slightest harm come from it.

For the pedicle I use No. 7, and smaller sizes for sewing and for adhesions. The catgut is prepared with ether to remove the oil. Then it is placed in a 1-500 sublimate solution for twenty-four hours, and later two or three days in a ten per cent. solution of juniper oil in alcohol, and is then preserved in alcohol until used.

Prepared in this way it is not very readily absorbed, is perfectly aseptic and reliable, and shrinks one-tenth of its length when wet with water. No. 7 will stand any strain which may be put on it.

I generally use the Staffordshire knot, putting the ends around the second time. The danger of secondary hemorrhage is imaginary. It is only necessary that a ligature should hold two or three days to ensure against secondary hemorrhage, as is conclusively proved by the clamp method in vaginal hysterectomy. When the clamp is taken off in thirty-six or forty-eight hours no hemorrhage results.

There is no proof that I have ever seen, that silk is ever absorbed. I have taken a piece out of the abdomen after it had been there a year. If it becomes infected at the bottom of a sinus it must be removed before the sinus will close, and to remove it, unless it comes away itself, is a matter of great difficulty and is often impossible.

I protest, then, most strongly against the discredit which is being thrown on catgut. If properly prepared, and you can only be sure of its proper preparation by doing it yourself, it is entirely without danger. The only case of secondary hemorrhage I ever met with, was from the slipping of a silk ligature, and never have I seen any harm which I could attribute to catgut.

I have now in the hospital a case not included in this report, in which it is the greatest satisfaction to me that I used catgut. I drained, and am sorry for it. I took out the tube in thirty hours, and when on the eighth day I went to remove the stitches, I found the hole where the tube had been open, and pus welling from it. The patient is doing well, but if I felt that there was a piece of infected silk at the bottom of that sinus, I should despair of ever seeing it close.[1]

I know of no more annoying complication of a laparatomy, or in fact of any gynecological operation, than an attack of cystitis. It sticketh closer than a brother, and often lasts to torment the patient for weeks after she is otherwise well. This trouble always arises from the use of a catheter, the instrument conveying septic matter from the outside, which sets up fermentation within, and consequent irritation. In order to prevent this infection of the bladder, I have recently adopted a plan, proposed in Germany I think, of using glass tubes for catheters, and having a little boric acid solution injected into the

1. Two knots of catgut came out of this sinus, after which it readily closed.

bladder each time before the catheter is withdrawn. In this way, it is claimed all danger of infection is done away with. This is a little point, but it is of great importance to the future comfort and welfare of the patient. The patient is also encouraged to get along without a catheter as soon as possible.

There are other sequelæ of laparatomy which are not so easily avoided. It has been my misfortune to see three of my patients become insane following the operation. Two of these cases were large ovarian tumors, while the third, after removal of diseased tubes and ovaries, recovered. I know of no way of avoiding this catastrophy. Still it must not be forgotten and must be thought of in estimating the chances, as a possible result in each case.

31 Allen Street.

---

